# Assessing neuromuscular system via patellar tendon reflex analysis using EMG in healthy individuals

**DOI:** 10.3389/fneur.2024.1522121

**Published:** 2025-01-30

**Authors:** Zakia Khatun, Sara Kristinsdóttir, Arndís Thóra Thórisdóttir, Linda Björk Halldórsdóttir, Francesco Tortorella, Paolo Gargiulo, Thordur Helgason

**Affiliations:** ^1^Department of Engineering, Institute of Biomedical and Neural Engineering, Reykjavik University, Reykjavik, Iceland; ^2^Department of Information and Electrical Engineering and Applied Mathematics, University of Salerno, Salerno, Italy; ^3^IT Development, Landspitali University Hospital, Reykjavik, Iceland; ^4^Development Department, Nox Medical ehf., Reykjavik, Iceland; ^5^Design Transfer, Össur ehf., Reykjavik, Iceland; ^6^Department of Science, Landspitali University Hospital, Reykjavik, Iceland; ^7^Department of Engineering, Reykjavik University, Reykjavik, Iceland

**Keywords:** electromyography, nerve conduction velocity, patellar tendon reflex, response time measurement, reference database

## Abstract

Patellar tendon reflex tests are essential for evaluating neuromuscular function and identifying abnormalities in nerve conduction and muscle response. This study explored how age, height, weight, and gender influence reflex response times in healthy individuals, providing a reference for future research on different neuromuscular conditions. We analyzed reflex onset, endpoint, and total duration of reflexes using electromyography (EMG) recordings from 40 healthy participants. Reflexes were elicited by striking the patellar tendon, and participants were grouped based on age, height, weight, and gender. We investigated both the individual and combined effects of these factors on reflex response times. Additionally, height and weight-normalized data were analyzed to clarify their roles in influencing reflexes across age groups. Gender-specific analyses were conducted as well to assess potential differences between males and females. Our findings indicated that reflex onset was significantly delayed in elderly individuals, particularly in taller and heavier individuals, and in males compared to females. Even with height normalization, elderly participants showed slower reflexes. Weight-normalized data revealed that younger participants exhibited longer total reflex durations, likely due to their greater height, which impacted nerve conduction time. This trend was consistent across genders, with males generally exhibiting longer duration of reflex response times. These findings provide insights into how different demographic factors, particularly aging, affect neuromuscular reflexes and could serve as a reference for diagnosing and monitoring neuromuscular disorders.

## 1 Introduction

**Figure 1:**
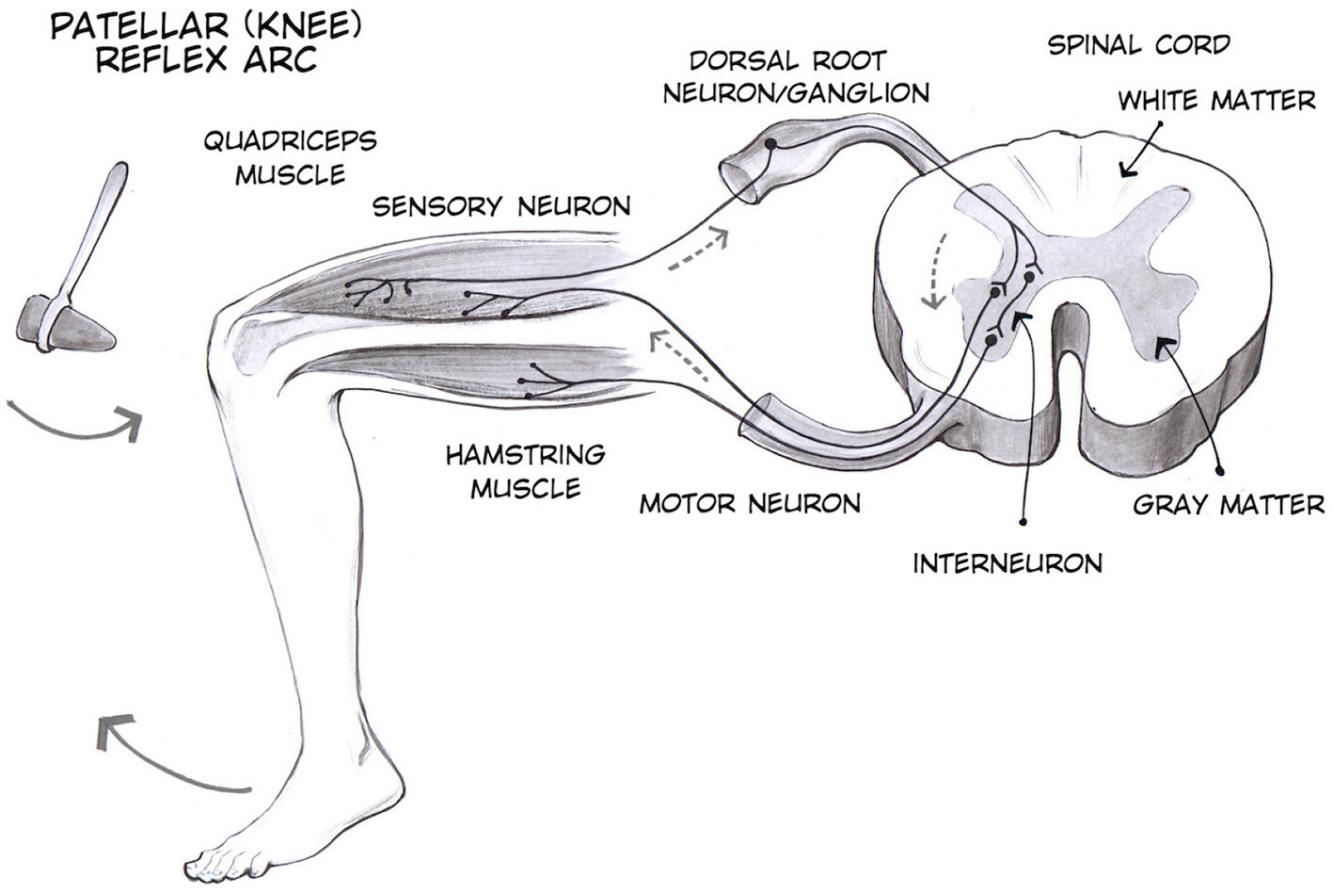
Patellar tendon reflex (3).

Tendon reflex is a standard component of neurological evaluation. The characteristics of the patellar tendon reflex (PTR) provide fundamental insight regarding the diagnosis of neurological status. The PTR is a deep tendon reflex that assesses the femoral nerve and spinal cord segments L2-L4 function. The PTR is elicited by tapping the patella tendon, causing the quadriceps to stretch. The stretch stimulates the muscle spindles, which leads to impulses being sent to the spinal cord via sensory afferents. The sensory fibers branch as they reach the spinal cord; some enter the gray matter of the cord and make monosynaptic contact with the lower motor neuron (LMNs), which stimulates the muscle to contract. As a result of this, the leg extends at the knee joint as the quadriceps muscle contract suddenly and the hamstring relaxes (1, 2). Due to the muscle contraction, the muscle spindles shorten again and their afferent activity is reduced (shown in Figure 1). Furthermore, sensory neurons act indirectly with interneurons to inhibit flexor motor neurons that would otherwise contract the opposite muscle, the hamstring.

This reflex is an essential tool for clinicians in evaluating the health and functionality of the nervous system. By observing and measuring the reflex responses, healthcare professionals can identify any abnormalities or changes that may indicate underlying neurological conditions. These reflexes are involuntary responses to stimuli, and alterations in their normal patterns can serve as early warning signs of potential issues such as nerve damage, neurological disorders, or systemic diseases. Therefore, careful analysis of reflex responses not only aids in diagnosing existing conditions but also plays a crucial role in monitoring the progression of neurological health over time (4).

The patellar tendon reflex is chosen for this study because it is commonly used to assess the integrity of the spinal cord and peripheral nervous system. This choice aligns with the broader goals of the two large-scale projects involved, which aimed to study both the spinal cord and tendons. The simplicity, reliability, and clear measurement of neural and muscular response times make the patellar tendon reflex ideal for examining how different demographic factors influence reflex timing.

Research has demonstrated that spinal reflexes vary significantly between younger and older individuals. Chandrasekhar et al. found that patellar reflex amplitude decreases with age, highlighting substantial differences in reflex magnitude across three age groups (5). Burke et al. further concluded that aging reduces reflex excitability, particularly during the Jendrassik maneuver, which involves clenching teeth and locking fingers while the patellar tendon is struck (6). Additionally, Koceja and Mynark revealed that older adults exhibit reduced monosynaptic reflex responses, particularly in standing positions, suggesting that age-related changes in the nervous system, such as motoneuron loss and decreased muscle spindle sensitivity, contribute to impaired balance and increased fall risk among the elderly (7). Carroll et al. investigated the *in vivo* mechanical properties of the patellar tendon, noting that differences related to aging are more associated with force output than direct age effects; however, decreased signal intensity indicated alterations in the internal milieu of the tendon, potentially affecting muscle spindle excitation during reflex hammer testing (8). Although Chandrasekhar et al. reported a significant age effect on the magnitude of patellar reflex responses, they found no influence of gender, concluding that neurologically normal individuals experience an age-dependent decline in patellar reflex. Notably, this study did not address whether the delay between the reflex hammer strike and the onset of EMG responses changes with age (5). Kamen and Koceja examined force-time characteristics of the patellar tendon reflex and found that older individuals produced greater overall reflex force; they also noted that a contralateral conditioning stimulus resulted in short-latency inhibition followed by longer-latency facilitation, both of which were more pronounced in aged subjects, indicating that age-related changes may occur at the spinal level and could influence the reaction delay of the quadriceps muscle following a reflex hammer strike (9). Hwang et al. investigated the impact of body weight on the soleus H-reflex and found that the amplitudes of the reflex significantly decreased as body weight load was reduced (10). Although this study did not examine response times, it highlighted the relationship between body weight and reflex amplitude. In the context of gender differences, one study utilizing surface electromyography indicated that males exhibit a slower patellar reflex compared to females (11). Supporting this, other studies have reported higher reflex responses in females (12). Conversely, some studies found no significant differences between the genders (13). As a result, the existence of sex differences in deep tendon reflexes remains a contentious topic in the literature (14). Campbell et al. examined the relationship between patient height and sex with patellar tendon length (PTL). They found no significant differences in mean age and body mass index between male and female patients, concluding that PTL is more strongly correlated with patient sex than with height. This suggests that the initiation of the patellar tendon reflex may differ between genders, while height appears to have a lesser impact (15). Pazzinatto et al. investigated women with and without patellofemoral pain (PFP) to compare the amplitudes of the patellar tendon reflex and the vastus medialis Hoffmann reflex (VM H-reflex) between the two groups. They found that women with PFP exhibited significantly lower amplitudes of the patellar T-reflex compared to pain-free controls, and that the VM H-reflex was strongly correlated with the patellar T-reflex in both groups (16). While there are numerous studies addressing tendon reflex responses, none specifically focus on how response times fluctuate when accounting for various demographic variables. This gap in the literature underscores the primary objective of our study.

This study investigates the neuromuscular system using the patellar tendon reflex test, specifically examining how age, height, weight, and gender influence reflex response times. To conduct this study, EMG data is collected using a surface EMG biofeedback system, and independent t-tests are performed to identify significant differences in response times between the groups.

## 2 Materials and methods

### 2.1 Hardware and software

KineLive is a wireless surface EMG biofeedback system (KISO) that has been shown to be an effective tool for diagnosing and treating problems with the human muscular-skeletal system (17). The wireless measurement units are small and light. The system consists of both a Kine measurements system and KinePro software. In this study, 4 and 8-channel EMG systems of KISO are utilized. The Kine unit amplifies the signal by 800, has a sampling frequency of 1,600 Hz, and a signal bandwidth of 16-500 Hz. The analog-to-digital converter (ADC) is 10-bit and the saturation level is ±2 mV. Each unit transfers the recorded EMG signal wirelessly to the measuring system, which is connected to a computer. The KinePro software then allows the recorded signal to be analyzed visually as well as transferred to other software applications for further research. The sEMG units are used with *Triode*^TM^ (T3402M) electrodes that have a 2 cm spacing of silver-silver chloride electrodes and nickel-plated brass snaps (18). The electrically synchronized reflex hammer used in this study includes an accelerometer to accurately measure the force and timing administered by the reflex hammer on the tendon of the subject. The accelerometer and its surrounding electronics are capable of accurately synchronizing with the EMG measurement with an error of less than 0.6 ms (19).

### 2.2 Participants

The dataset used in this study comprises 40 participants with informed consent, categorized into two distinct age groups. The younger cohort comprises 25 individuals, with an average age of 23.76 ± 1.75 years, an average height of 177.48 ± 12.52 cm, and an average weight of 75.28 ± 16.17 kg. This group includes 14 males and 11 females. In contrast, the elderly cohort comprises 15 individuals, with a mean age of 59.8 ± 5.66 years, an average height of 174.33 ± 6.51 cm, and an average weight of 82.33 ± 13.68 kg, including 8 males and 7 females. Participants are selected based on predefined health criteria, ensuring that all individuals are in good health at the time of data collection. This selection process ensured that the dataset would reflect typical physiological characteristics without the influence of significant underlying health conditions, providing a more accurate baseline for the focus of this study. This database serves as a reference collection of healthy cases and can be used as a baseline for studying neurological disorders.

The physiological measurements were approved by the Medical Director of Landspítali, the Science Ethics Committee, and the Icelandic Data Protection Act. The Icelandic name of the ethics committee is Vísindasiðanefnd (The national bioethics committee, in English), under permission no. 14-150-S1, with an approval date on November 18, 2014. The study title in Icelandic is *Raförvun mænu með yfirborðsrafskautum, færnibætandi áhrif á s*í*spennu* (translated to English: Transcutaneous spinal cord stimulation for skill-improving effects on spasticity).

**Figure 2:**
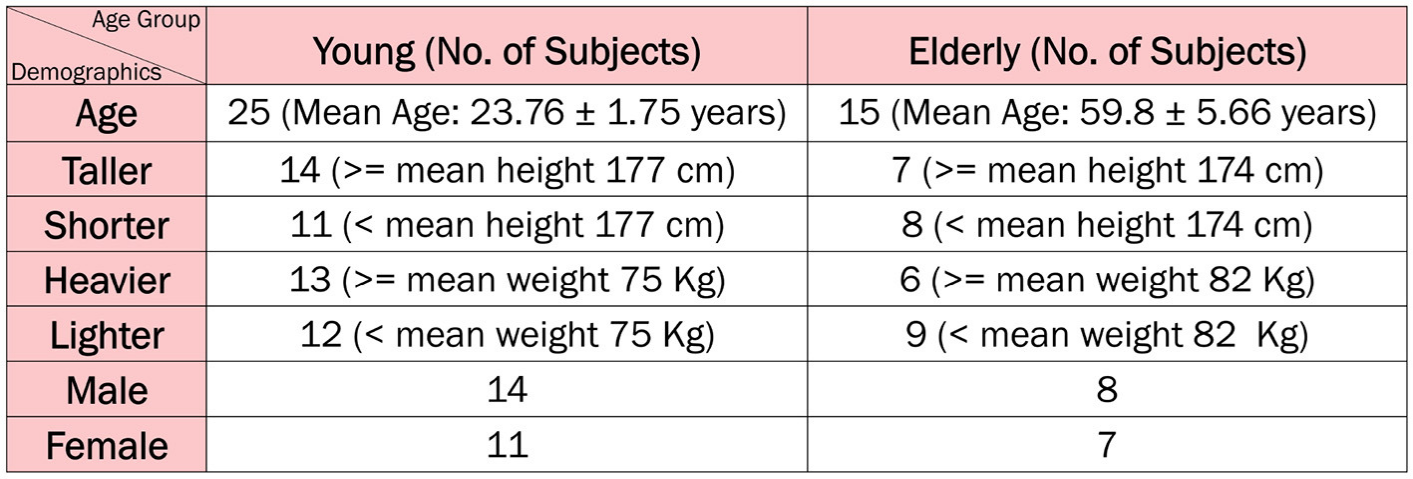
Study population.

Figure 2 illustrates the distribution of subjects across various categories and age groups.

### 2.3 Data acquisition protocol

**Figure 3:**
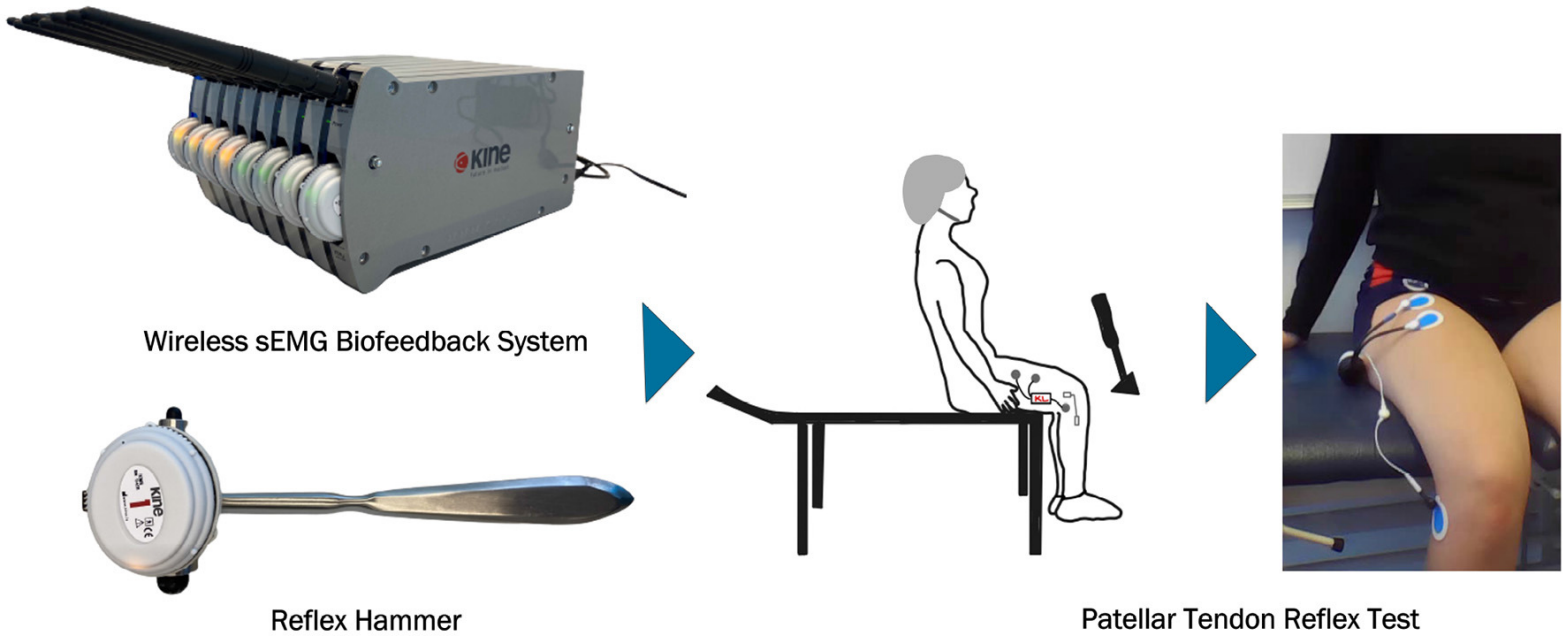
Data acquisition.

The data acquisition protocol used in our study is illustrated in Figure 3, which depicts the electrode placement and overall setup. The detailed steps of the protocol are described below.

1. Administer a questionnaire to collect the subject's age, height, and weight. The questionnaire also records any known underlying illnesses or injuries, the frequency of physical activity per week, and consumption of alcohol and caffeine.
2. Have the subject sit on a bench with their legs hanging freely without touching the ground.
3. Verify that the subject is not wearing any electronic devices to minimize background noise.
4. Mark the appropriate locations for surface electrode placement. The first EMG electrode (anode) is positioned approximately one-third of the distance from the subject's hip joint to the upper edge of the patellar bone. The second EMG electrode (cathode) is placed 6 cm below this point.
5. Adjust the final electrode placement to ensure they are positioned over the rectus femoris for accurate reflex response measurement.
6. Cleanse the skin with alcohol and apply conductive gel to enhance electrical conductivity before placing the EMG electrodes.
7. Attach the EMG electrodes to the skin using KineLive sEMG units to collect EMG data.
8. Place a grounding electrode on the head of the tibia bone.
9. Use a second KineLive sEMG unit integrated into a medical hammer to measure the force applied to the patellar tendon and synchronize this data with the reflex EMG data.
10. Connect the KineLive sEMG units to the KinePro software for synchronization.
11. Initiate the recording by clicking the record measurements button in the software, with the recording lasting a total of 5 min.
12. During the 5-minute recording, strike the patellar tendon with the reflex hammer in about every 15–20 s while recording the EMG data in KinePro software. Ensure the subject remains still, calm, and blindfolded to prevent anticipation of the strikes and ensure unbiased reflex data.

To reduce variability in strike localization, the midpoint of the patellar tendon is pre-marked to ensure consistent and accurate targeting. This pre-marking helps to eliminate guesswork and enhances precision during the procedure. Additionally, to standardize the force and direction of the strike, the examiner's elbow is supported, providing stability and enabling controlled, precise wrist movements.

### 2.4 Data analysis

**Figure 4:**
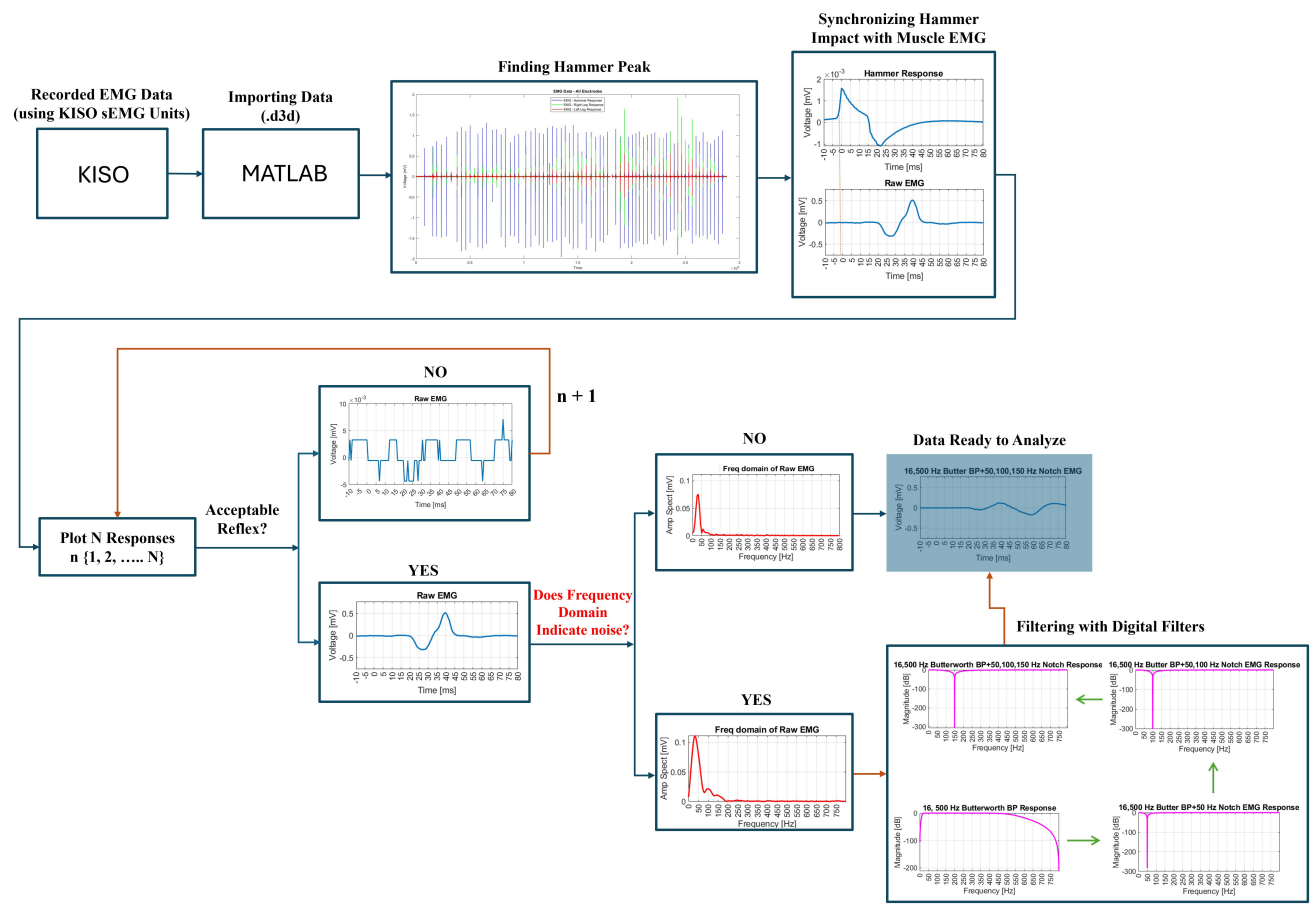
Pipeline to select impactful signals only.

The pipeline of this study, illustrated in Figure 4, is designed to select only impactful signals for the final data analysis. This pipeline is implemented using Matlab R2023b. A portion of this pipeline was developed and used in a previous study with different objectives (20).

Initially, the recorded data (.d3d format) are imported into the Matlab environment. The first step involves identifying and extracting the hammer impact or impulse peaks to synchronize them with the corresponding electromyography (EMG) signals. This synchronization enabled the segmentation of the EMG data into individual epochs, where each epoch represented the EMG response to a specific hammer stimulus. These epochs are plotted for visual inspection to confirm accurate segmentation. Next, each EMG response to the hammer impact is carefully reviewed to assess whether it contains an acceptable reflex or not. This process involved identifying a clear reflex response with physiologically plausible latency, distinct activation and relaxation phases, and consistent amplitude. Signals with excessive noise, irregular patterns, or implausible latencies were excluded from the analysis. Figure 4 illustrates examples of acceptable and unacceptable reflex responses based on these criteria. The remaining acceptable reflexes are then checked for digital noise to ensure their reliability for further analysis. Any responses exhibiting excessive noise or deemed insufficient for detecting critical time points, such as latency, are excluded. Valid, noise-free responses are retained in a new dataset for further processing, including frequency domain analysis.

During the frequency domain analysis, any interference at 50 Hz, commonly associated with power line noise, is addressed by applying a 50 Hz notch filter. This filter effectively eliminated the 50 Hz interference. Prior to this step, a 4th-order Butterworth bandpass filter, with a lower cutoff frequency of 16 Hz and an upper cutoff frequency of 500 Hz, is applied to the EMG data. This bandpass filter helps to isolate the relevant signal components by removing unwanted low-frequency and high-frequency noise. This 4th-order Butterworth bandpass filter is applied using the filtfilt() function in Matlab (21). This method helps to eliminate phase distortion, ensuring precise timing between the stimulus and response, which is critical for accurately assessing reflex response latencies and durations. In cases where additional noise at 100 Hz or 150 Hz is detected, corresponding notch filters are applied to eliminate this specific interference. The effectiveness of each digital filter is validated by examining the frequency and magnitude responses in the frequency domain. Once filtering is complete, the data is deemed suitable for the final analysis.

### 2.5 Response time analysis across subject groups

**Figure 5:**
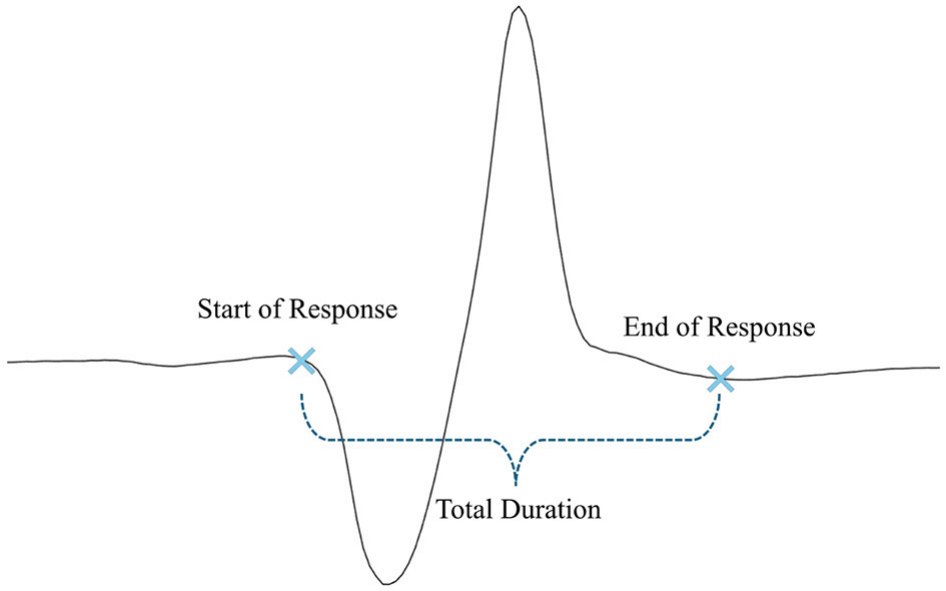
Measured response times.

Following the data processing, the response times of different subject groups are analyzed. Response times are measured in terms of the start of the response, the end of the response, and the total duration (illustrated in Figure 5).

The start of the response is defined as the precise moment when the reflex is triggered, indicating the onset of muscular activity following the stimulus. The end of the response marks the point when the muscle activity ends. The total duration represents the time from the initial reflex trigger to the complete relaxation of the muscle. These metrics provide valuable information about the efficiency and speed of the neuromuscular response. After processing, these response times are analyzed across various subject groups to identify patterns or differences in reflex behavior based on factors such as age, height, weight, and gender. The specific groups studied in this study are outlined in the following sections.

1. **Age group only**: The response times of elderly versus young individuals are analyzed. Elderly individuals are defined as those over 50 years of age, while young individuals are defined as those younger than 30 years of age. These age thresholds are selected based on the characteristics of our study population, ensuring clear distinctions between younger and older participants. The aim here is to compare the response times between these two distinct age groups.
2. **Age + height group**: In this experiment, the effect of height on response times within different age groups is examined. For elderly individuals, the mean height is calculated to be 174 cm. Elderly individuals whose height is greater than or equal to this value are labeled as taller, while those shorter than the mean are labeled as shorter. For the young group, the mean height is 177 cm. Young individuals taller than or equal to this height are considered taller, while those shorter than the mean are considered shorter. The response times are then compared between taller elderly versus taller young, and shorter elderly versus shorter young, to determine whether height significantly influences response times across age groups or not.
3. **Age group only (height normalized)**: Since individual heights vary, the response times are normalized by dividing the recorded times by each subject's height to account for height differences. This normalization allows for a more consistent comparison of response times across individuals, independent of height.
4. **Age + weight group**: This experiment examines the impact of weight on response times in different age groups. For elderly individuals, the mean weight is 82 kg. Elderly individuals who weigh more than or equal to the mean are labeled as heavier, others as lighter. Similarly, for younger individuals, the mean weight is 75 kg. Young individuals weighing more than or equal to the mean are marked as heavier, others as lighter. The response times of heavier versus lighter individuals within each age group are then compared.
5. **Age group only (weight normalized)**: To eliminate bias caused by varying weights, the response times are normalized by dividing each subject's response time by their respective weight. This normalization removes the influence of weight, allowing for an unbiased analysis of response time differences across individuals.
6. **Age + gender group**: In this setup, the effect of gender on response times within each age group is analyzed. Elderly males are compared to young males, and elderly females are compared to young females, to investigate potential gender-based differences in response times across age groups.
7. **Gender group only**: In addition to age-related comparisons, this experiment focuses exclusively on the effect of gender. Response times are analyzed by comparing all male individuals versus all female individuals, regardless of age, to assess whether gender alone has a significant impact on response times.

## 3 Results

### 3.1 Age group only

The comparison of response times between elderly and young individuals reveals a significant difference in the start of response (*p* = 0.007). The elderly individuals exhibits a slower onset time, with the response starting at 20.92 ± 3.013 ms, compared to 17.68 ± 3.708 ms for the young group. The end of response shows a marginal difference (*p* = 0.068), with the elderly group completing the response slightly later (78.47 ± 1.995 ms) compared to the young individuals (77.08 ± 2.397 ms), although this difference is not statistically significant. Additionally, the total duration of the reflex shows no significant difference between the two groups, with elderly individuals recording a duration of 57.55 ± 2.901 ms and young individuals 59.40 ± 4.013 ms, reflected by a p-value of 0.127.

**Figure 6:**
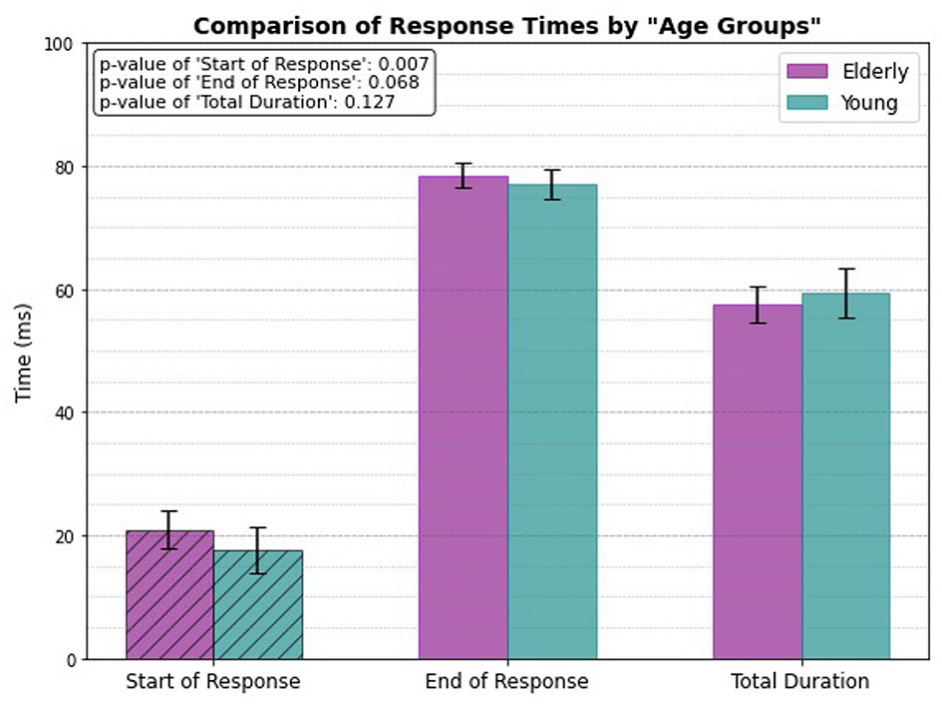
Only age related response.

Figure 6 presents the response times of the two age groups, with dashed bars used to highlight the response times that show a statistically significant difference (specifically, the start of response in this case).

### 3.2 Age + height group

**Figure 7:**
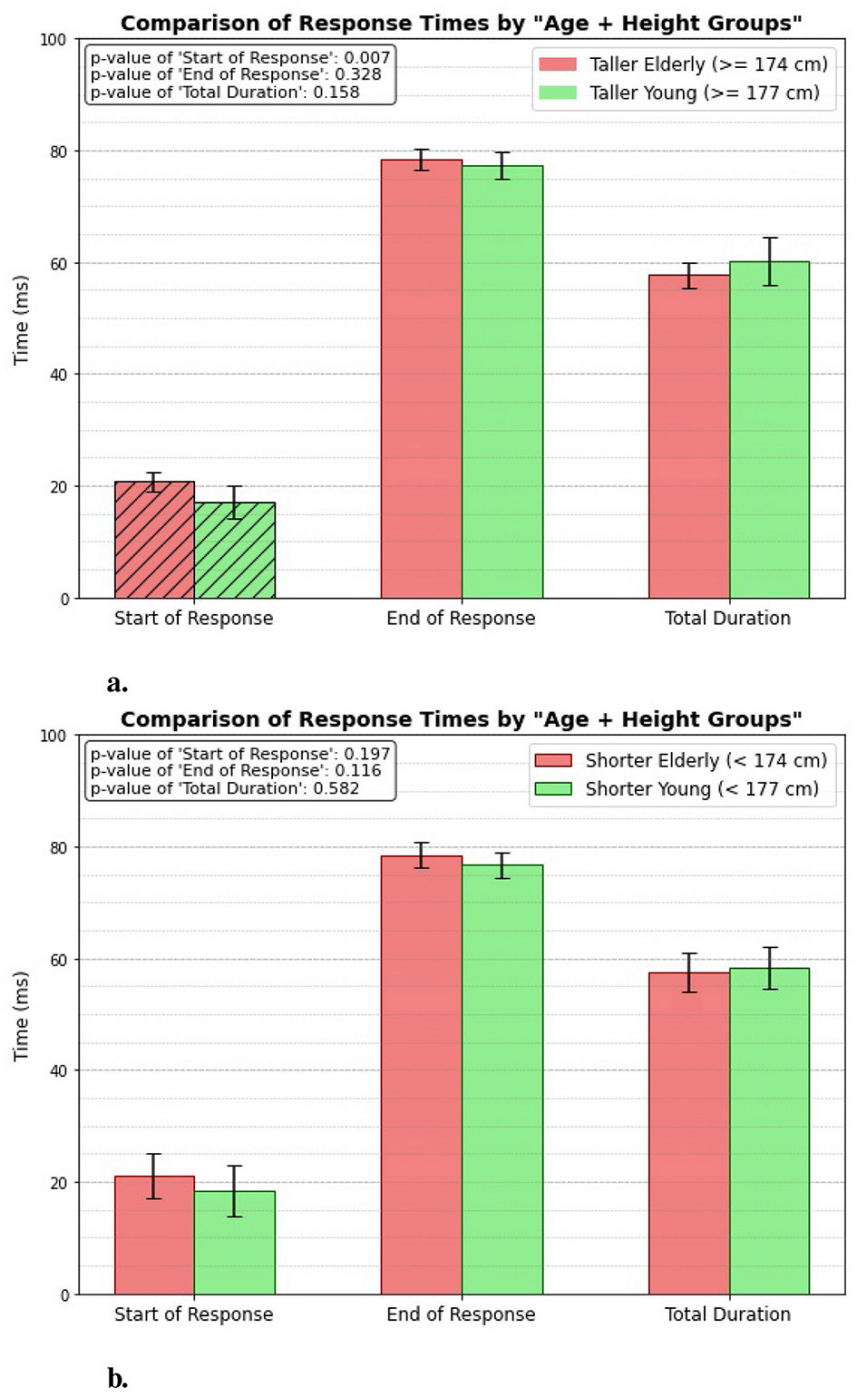
Age + height related response. **(A)** Taller elderly vs. taller young. **(B)** Shorter elderly vs. shorter young.

The response times between the two groups, Taller Elderly (≥174 cm) and Taller Young (≥177 cm) (Figure 7A), shows that the taller young group gets reflex triggered significantly much faster (p = 0.007). The mean value of start of response for the taller young group is 17.14 ± 2.898 ms, compared to 20.74 ± 1.656 ms for the elderly group. However, both groups exhibit similar times for the end of the response, with no significant difference (p = 0.328). The total duration also shows no significant variation between the groups (p = 0.158).

Figure 7B presents the measured response times for the Shorter Elderly (height < 174 cm) and the Shorter Young (height < 177 cm) groups. The results indicate that there is no significant difference between the groups in terms of the start of response, end of response, or total duration of the patellar tendon reflex. This lack of significant variation suggests that height does not have a considerable impact on reflex response times among shorter individuals, regardless of age.

### 3.3 Height normalized, age group only

**Figure 8:**
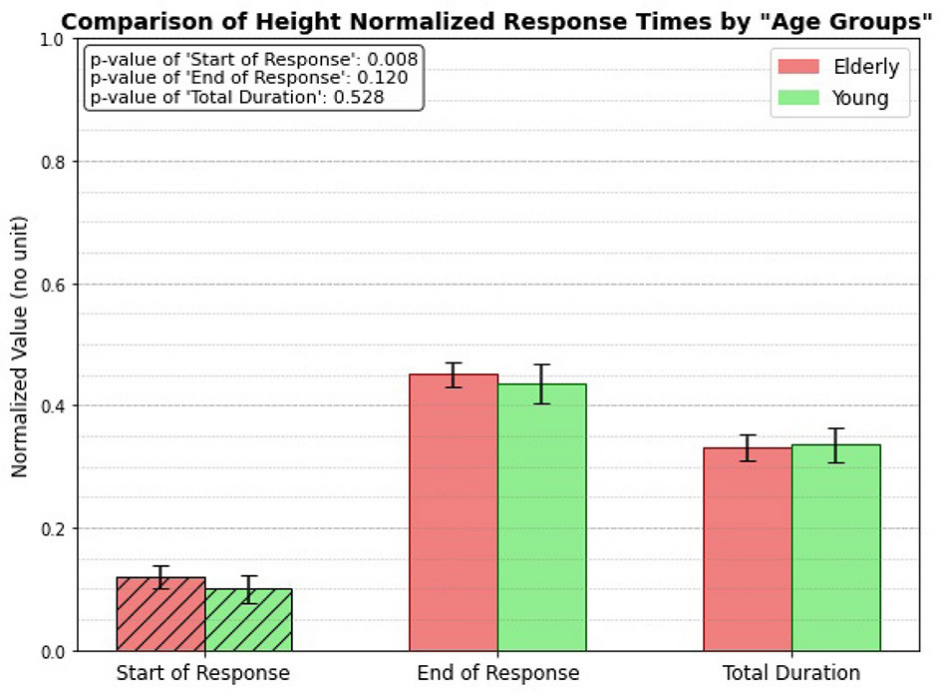
Height normalized, age related response.

Figure 8 illustrates the height-normalized response times (unitless, since divided by heights) for both elderly and young individuals. The bar plots clearly demonstrate that there is a significant difference in the start of response between the two groups, with a p-value of 0.008. However, the analysis reveals no significant differences in the end response or total duration categories between the elderly and young individuals. This suggests that height normalization influences the timing of the reflex initiation but does not affect the other aspects of the response.

### 3.4 Age + weight group

**Figure 9:**
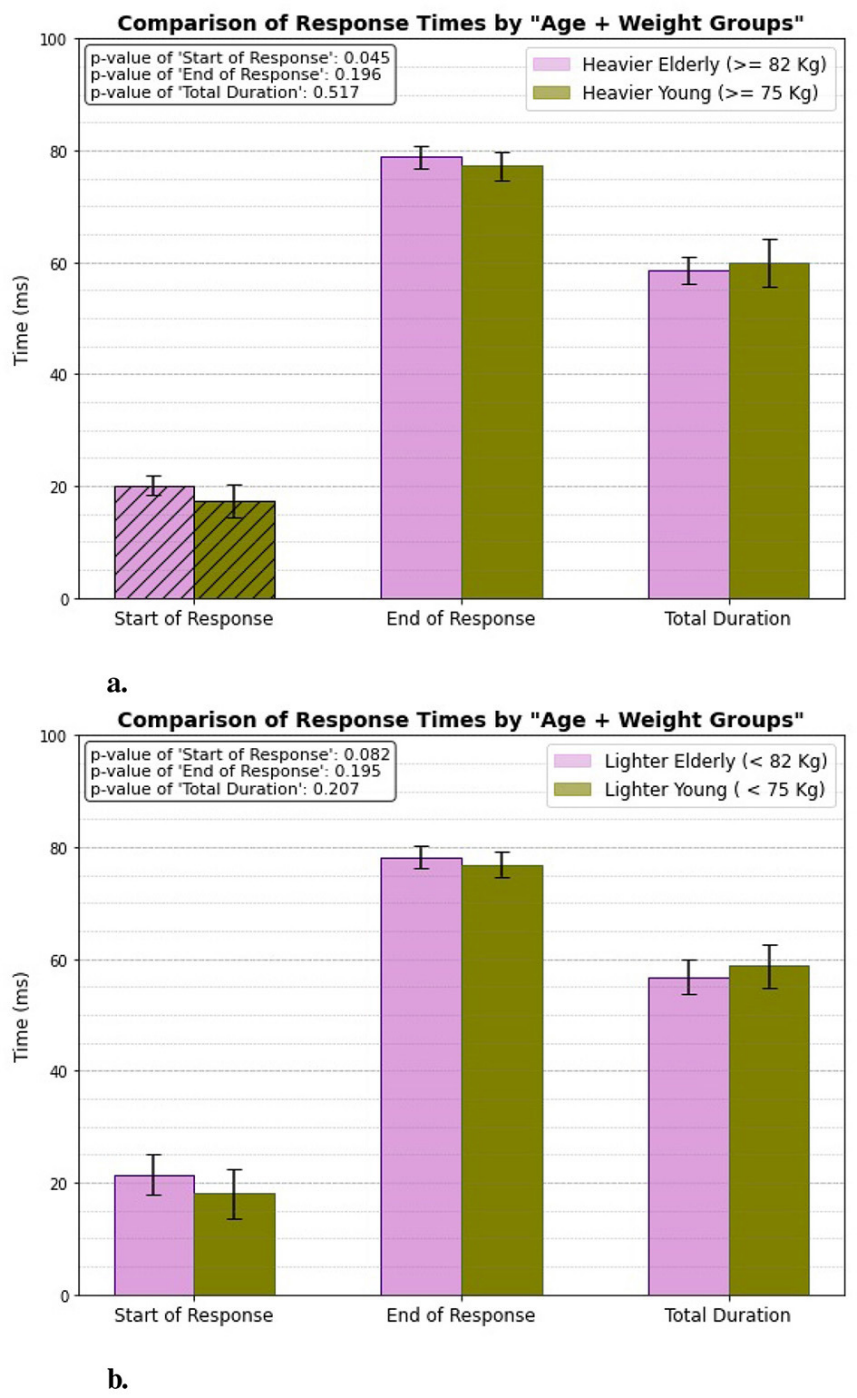
Age + weight related response. **(A)** Heavier elderly vs. heavier young. **(B)** Lighter elderly vs. lighter young.

Figure 9 illustrates how aging influences patellar tendon reflex response times, particularly in relation to the individuals' weight. The results indicate a significant difference in the start of response times, highlighting that weight plays a role in the initiation of the reflex response. Specifically, the heavier individuals exhibit a quicker start to their reflex action. The start of response occurs at 17.3 ± 2.947 ms for the heavier young individuals, while it is measured at 20.14 ± 1.742 ms for the heavier elderly individuals.

However, for the lighter individuals, the difference in response times is less pronounced, suggesting that while weight impacts reflex initiation, the effect is not as strong in this group. In contrast, when evaluating the end of response and total duration, there are no significant differences observed between either the heavier or lighter groups. This finding suggests that, while weight may influence the speed of reflex initiation, it does not significantly affect the overall duration or the termination of the patellar tendon reflex response in either age group.

### 3.5 Weight normalized, age group only

**Figure 10:**
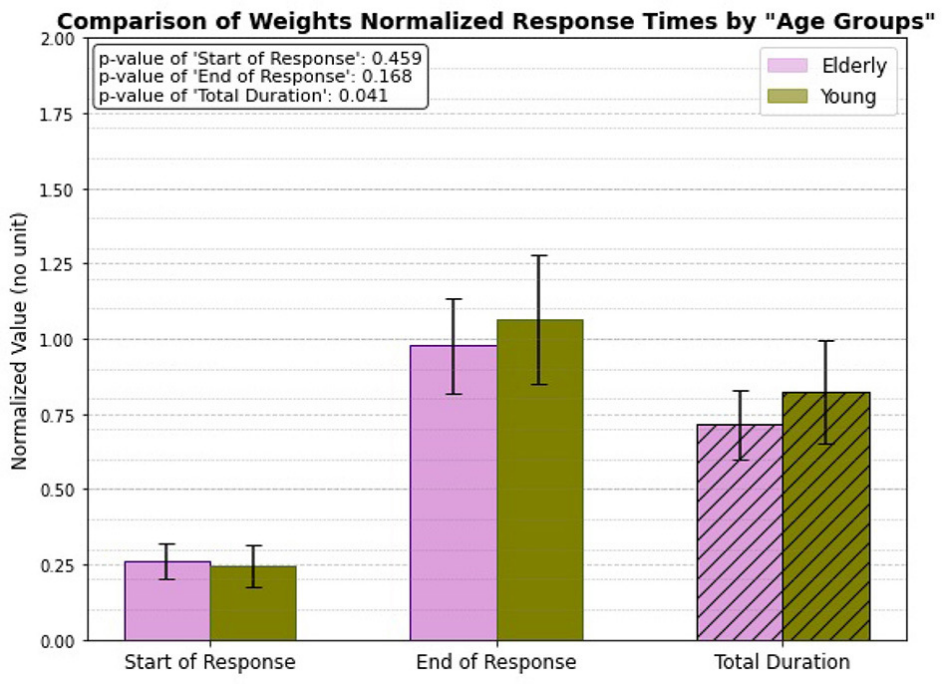
Weight normalized, age related response.

When analyzing the weight-normalized response times, a significant difference emerges between the elderly and young subject groups in the total duration of muscle activation (illustrated in Figure 10). The total duration for the young individuals, after normalizing for weight, is noticeably longer compared to that of the elderly individuals, with a p-value of 0.041 indicating statistical significance. However, no significant differences are observed in the start of response and end of response times between the two groups. This suggests that while the overall duration of muscle activation is influenced by age in the context of weight normalization, the initiation and ending of the patellar tendon reflex response remain similar across both young and elderly individuals.

### 3.6 Age + gender group

**Figure 11:**
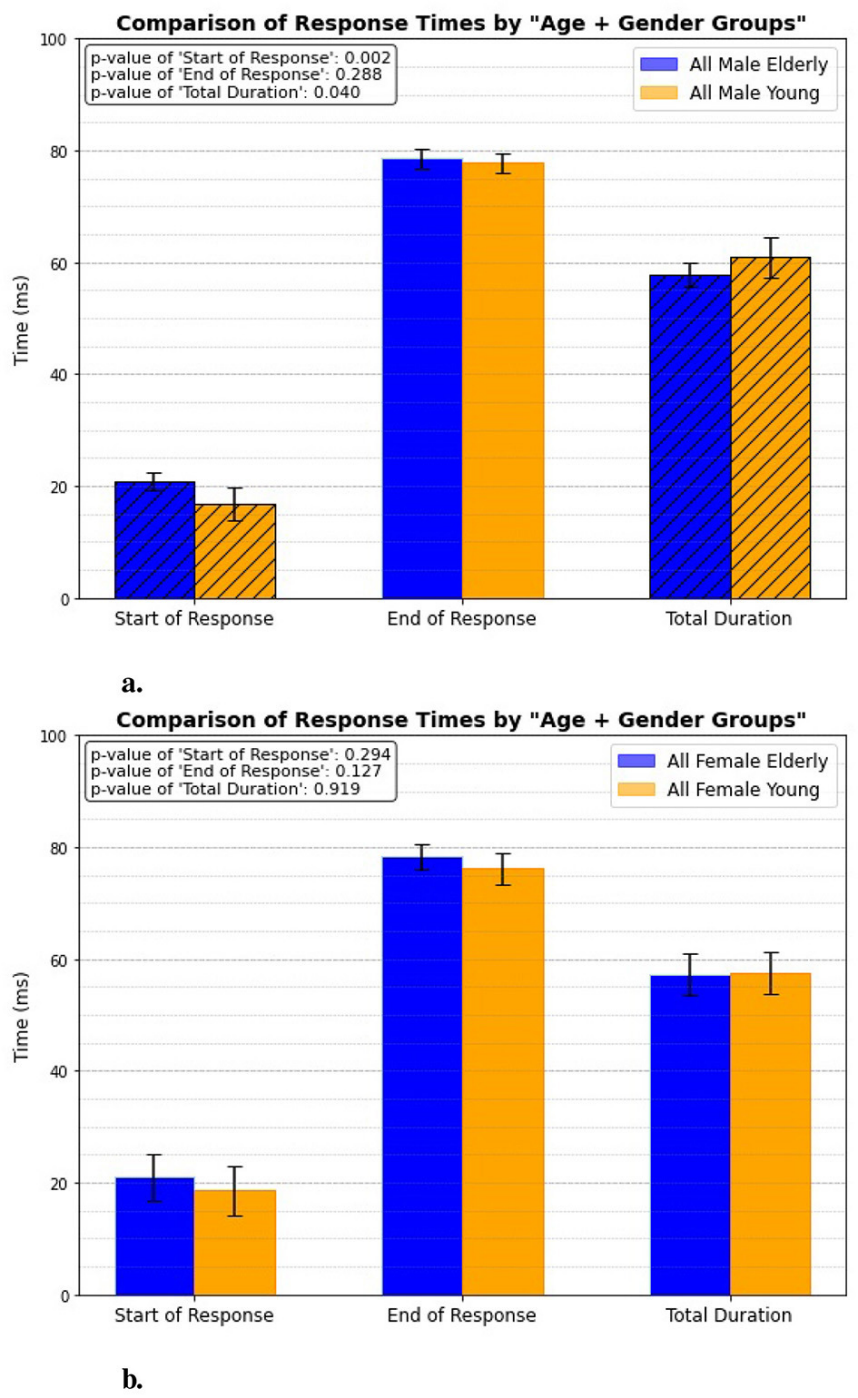
Age + gender related response. **(A)** All male elderly vs. all male young. **(B)** All female elderly vs. all female young.

In this case, the response timings are analyzed for both elderly and young individuals, with gender being a key factor. One experimental category focuses exclusively on males from both age groups, as shown in Figure 11A, while another category examines all females from the same age groups, illustrated in Figure 11B. The results reveal a significant difference in response timings only among males, with p-values of 0.002 for the initial activation and 0.04 for the overall total duration. This suggests that male individuals exhibit notable variations in both the speed of reflex initiation and the length of the reflex response compared to their female group. However, when examining the female individuals, the analysis shows no significant differences in response timings between the elderly and young groups.

### 3.7 Gender group only

**Figure 12:**
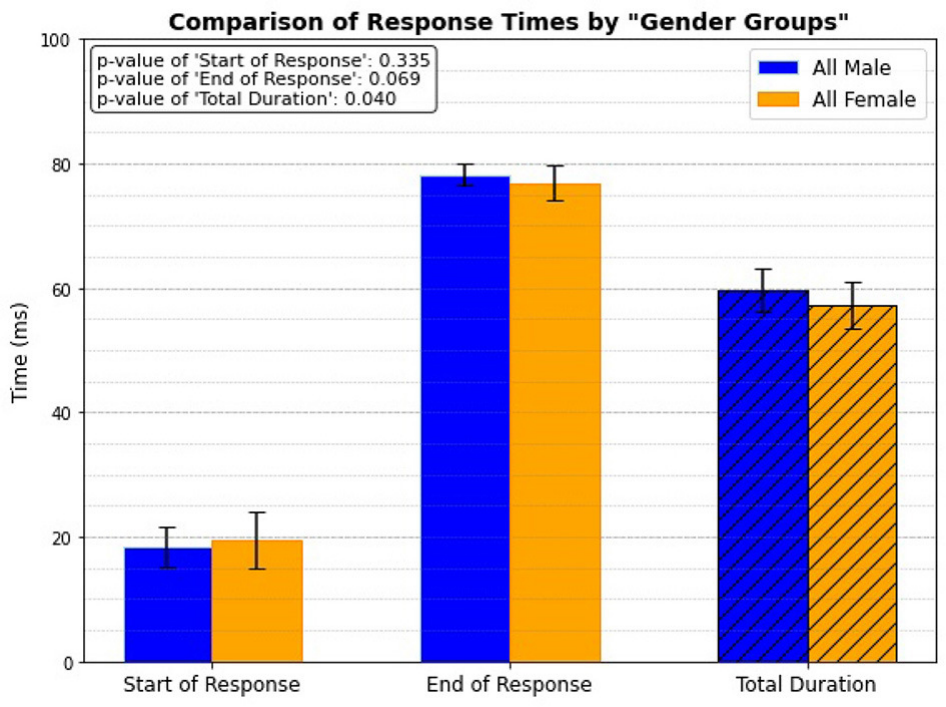
Gender related response.

Lastly, the study focuses exclusively on gender groups, regardless of age, to investigate if there is any significant differences in reflex response timing. The results indicate that males, regardless of whether they are elderly or young, exhibit longer response times, with a statistical significance of p = 0.04 (Figure 12). This finding aligns with the data presented in Figure 11A, which also considers age as a factor.

## 4 Discussion

### 4.1 Age group only

The PTR test reveals notable differences in response times between elderly and young individuals, particularly at the onset of the reflex response. A significant delay in onset time (p = 0.007) is observed in the elderly group, suggesting that aging slows neural processing and motor response initiation. This delay is likely due to age-related physiological changes, such as decreased nerve conduction velocity, reduced muscle mass, and declined neuromuscular efficiency. Burke et al. indicated that aging reduces reflex excitability, particularly during the Jendrassik maneuver (6). Further research has shown that older adults exhibit diminished monosynaptic reflex responses, especially in standing positions (7). Overall, our results are consistent with the literature on age-related changes in reflex responses. In contrast, younger individuals generally have a higher proportion of fast-twitch muscle fibers, which may contribute to a quicker onset time. Interestingly, still the total duration of the reflex remains somewhat similar between this two groups. This suggests that while the initiation of the reflex is slower in the elderly, the overall reflex duration stays comparable. Elderly individuals may rely on compensatory mechanisms, such as greater reliance on experience or adaptations to physiological changes. According to Ward, the recruitment of additional motor regions in elderly adults provides a compensatory mechanism to counteract age-related brain changes (22). Similarly, studies by Takeuchi et al. and Goble et al. highlighted how overactivation of specific brain regions, such as the prefrontal cortex and supplementary motor areas, may compensate for declines in motor control and cognitive tasks in older adults (23, 24). Furthermore, Reuter-Lorenz et al. suggested that the decreased lateralization in older adults could be compensatory, reflecting the recruitment of additional brain regions to support task performance (25). For instance, although nerve conduction is slower, the efficiency of muscle contractions and movement coordination remains intact, allowing the reflex to occur within a similar timeframe as in younger individuals.

### 4.2 Age + height group

When categorizing individuals by height in addition to age, a similar pattern emerges where the elderly group shows a delayed onset of reflex response compared to the younger group, but there is no significant difference in the end of the reflex or the total duration. However, this pattern is significant only among taller individuals. In contrast, for shorter individuals, no significant differences are observed between the elderly and young groups in any of the reflex measures. The lack of significant variation in reflex response times between the shorter elderly and shorter young groups suggests that height does not play a major role in reflex timing for shorter individuals, regardless of age. This may be because the neural and musculoskeletal distances involved in the patellar tendon reflex arc (i.e., the pathway from the muscle spindles to the spinal cord and back) are relatively consistent among shorter individuals. Since the speed of the reflex depends on how quickly signals travel through nerves and how fast muscles respond, the shorter distances in these individuals likely lead to less variability in reflex times. In contrast, taller individuals have longer neural pathways, which may introduce more variability in reflex timing between age groups. This is consistent with the findings of Péréon et al., which demonstrated that there is a strong correlation between height and latency - specifically, as height increases, so does latency (26). However, for shorter individuals, these distances are smaller and less variable.

### 4.3 Height normalized, age group only

Given the previous experimental outcomes, the objective of this step was to determine whether height impacts the performance differences between age groups. The results show that regardless of whether response times are analyzed based on height categories (taller vs. shorter) or height-normalized, the onset of the reflex is consistently delayed in elderly individuals (*p* = 0.008). However, no significant differences are observed in the end response or total reflex duration between the two age groups. This suggests that while the initiation of the reflex is slower in the elderly, the overall duration remains comparable. This is likely due to compensatory mechanisms and adaptations to physiological changes, as discussed earlier. These adaptations may allow elderly individuals to maintain a similar total reflex time, despite the delayed onset.

### 4.4 Age + weight group

In studying the combined effect of age and weight, we observe the familiar pattern that elderly individuals have slower reflex onset times compared to younger ones, though there is no significant difference at the end of response or total duration. However, two interesting observations emerge from this weight-related analysis. First, both elderly and young individuals in the heavier group demonstrate quicker reflex onset times than their lighter counterparts. This difference may be attributed to the greater muscle mass and increased mechanical load in heavier individuals, which can enhance motor responses and sensitivity in reflex pathways. Second, the expected age-related delay in reflex onset occurs only in the heavier individuals. Among lighter individuals, there is no significant difference in response times between elderly and young individuals. This could be because lighter individuals experience less mechanical stress, which minimizes the impact of weight on their reflex performance. Additionally, lighter individuals typically experience less strain on their muscles and joints due to their lower body weight. This reduced mechanical load during reflex actions contributes to more consistent reflex performance across different age groups, as lighter individuals are less affected by the declines in muscle strength and coordination associated with aging.

### 4.5 Weight normalized, age group only

The findings indicate a significant difference in the total duration of muscle activation during the weight-normalized patellar tendon reflex between young and elderly individuals, with young individuals demonstrating longer activation times (*p* = 0.04). This difference may be attributed to age-related changes in muscle fiber composition and recruitment patterns. Additionally, the higher height of younger individuals could influence the time required for neural signals to travel between the muscle spindles to the spinal cord and back, potentially contributing to the longer activation duration. Although, there are no significant differences observed in the onset and end times of the reflex between the two groups.

### 4.6 Age + gender group

The results from the patellar tendon reflex test suggest that aging significantly affects reflex response times in males but not in females, highlighting a gender-specific difference in neuromuscular aging. In contrast, the absence of significant differences in all response times for females (*p*-values >0.1) indicates that their reflex response times remain stable with age. This gender disparity could be attributed to biological differences, including hormonal factors, or potentially different patterns of physical activity and muscle health across the lifespan. These findings imply that age-related neuromuscular decline may be more pronounced in males.

### 4.7 Gender group only

The analysis of reflex response timings across gender groups, excluding age considerations, indicates that males exhibit significantly longer total response times than females (*p* = 0.04). This finding implies that, independent of aging effects, males may possess longer neuromuscular reflexes. One potential explanation for this difference is the average height of males compared to the mean height of females. Taller individuals typically have longer nerve pathways, potentially extending the time required for signals to travel between the muscle spindles to the spinal cord and back, which is involved in reflex actions. Additionally, greater limb length and increased muscle mass in males may necessitate more time for the neuromuscular system to fully activate, further contributing to the observed differences in reflex response times. While the differences in the start and end of responses are not statistically significant, the prolonged total duration in males suggests a more extended reflex action overall.

## 5 Conclusion

This study has established a quantitative baseline for interpreting patellar tendon reflex (PTR) responses by analyzing factors such as age, height, weight, and gender in healthy individuals. While physicians typically rely on qualitative assessments of PTR for general diagnostic purposes, our approach provides an objective and quantitative method for evaluating neuromuscular reflexes. The results offer a benchmark for assessing typical functional profiles in healthy individuals, which can serve as a reference point for future studies, enabling comparisons with pathological cases and aiding in the detection of subtle abnormalities. By distinguishing normal age-related changes from pathological variations, our findings pave the way for improved diagnostic accuracy and the development of advanced therapeutic tools in neuromuscular medicine.

Our study demonstrates that aging is linked to delayed reflex response onset in elderly individuals, which supports established theories about age-related physiological changes. While elderly individuals exhibit slower reflex initiation, the overall duration of the reflex remains comparable to that of younger individuals. Additionally, the interaction between age and height shows that elderly individuals experience pronounced delays in reflex onset, particularly among taller individuals, with shorter individuals not demonstrating this delay. Even when the data is height normalized, the pattern of delayed onset persists, indicating that, regardless of height considerations, younger individuals consistently show faster onset times than their elderly counterparts. In terms of the impact of weight, our study reveals that elderly individuals generally respond more slowly to reflex tests, particularly among heavier individuals. Additionally, when weight normalization is applied to assess the aging effect, younger individuals demonstrate longer reflex completion times, which correlates with their height, as taller individuals would naturally require more time to complete the reflex response. The study also reveals gender-specific differences in reflex response times, highlighting that aging impacts males more significantly than females. This suggests that biological and physiological factors may play a role in neuromuscular aging, leading to the observed differences in reflex responses. Furthermore, when considering only gender groups, males exhibit a longer total reflex duration, likely due to their generally greater height, which necessitates more time for signals to travel the longer nerve pathways.

Despite providing valuable insights, this research has a few limitations as well. The small sample size may limit the generalizability of the findings, and the cross-sectional data collection hinders the establishment of causal relationships. Expanding research to include more diverse populations could deepen our understanding of age-related neuromuscular decline and the influence of gender and body mass index. This study serves as an exploratory analysis, generating preliminary insights. To address its limitations, including the use of independent t-tests with increased Type I error risk and challenges with small sample sizes, future work will involve re-analyzing the data using more robust methods, such as ANOVA test, on a larger cohort. This will allow us to perform experiments on more than two independent groups to study the effect of multiple demographic factors simultaneously. In addition, despite the efforts to standardize the procedure of strikes, the manual nature of the process introduces inevitable variability, which remains a limitation of this study. Future research could use automated or instrumented reflex hammers to minimize these variations and improve the consistency of reflex recordings. This may pave the way for improved diagnostic tools and more effective therapeutic strategies.

## Data Availability

The dataset presented in this article is not readily available because we are working on enlarging it, thus making it statistically more reliable, and it has not yet been decided whether or when the dataset will be made public. Requests to access the datasets should be directed to thordur@landspitali.is.

## References

[1] Pocock G Richards CD. The Human Body: An Introduction for the Biomedical and Health Sciences, 1st ed. Oxford: Oxford University Press. (2009).

[2] Noback CR. The Human Nervous System: Structure and Function, 6th ed. Totowa, NJ: Humana Press. (2005).

[3] Brains B. Muscle Knee Jerk Reflex. (2024). Available at: https://backyardbrains.com/experiments/Musclekneejerk#prettyPhoto (accessed 13 December, 2024).

[4] Kandel ER Schwartz JH Jessell TM Siegelbaum SA Hudspeth AJ. Principles of Neural Science, 5th ed. New York, NY: McGraw-Hill Education (2013).

[5] Chandrasekhar A Abu Osman NA Tham L Lim KS Abas W. Influence of age on patellar tendon reflex response. PLoS ONE. (2013) 8:e80799. 10.1371/journal.pone.0080799 24260483 PMC3832481

[6] Burke JR Schutten MC Koceja DM Kamen G. Age-dependent effects of muscle vibration and the Jendrassik maneuver on the patellar tendon reflex response. Arch Phys Med Rehabil. (1996) 77:600–4. 10.1016/S0003-9993(96)90302-0 8831479

[7] Koceja DM Mynark RG. Comparison of heteronymous monosynaptic Ia facilitation in young and elderly subjects in supine and standing positions. Int J Neurosci. (2000) 103:1–17. 10.3109/00207450009035005 10938558

[8] Carroll CC Dickinson JM Haus JM Lee GA Hollon CJ Aagaard P. Influence of aging on the in vivo properties of human patellar tendon. J. Appl. Physiol. (2008) 105:1907–15. 10.1152/japplphysiol.00059.2008 18927271 PMC2612460

[9] Kamen G Koceja DM. Contralateral influences on patellar tendon reflexes in young and old adults. Neurobiol Aging. (1989) 10:4. 10.1016/0197-4580(89)90041-9 2682314

[10] Hwang S Jeon HS Kwon OY Yi CH. The effects of body weight on the soleus H-reflex modulation during standing. J Electromyog Kinesiol. (2011) 21:445–9. 10.1016/j.jelekin.2010.11.002 21144768

[11] Lin-Wei O Xian LLS Shen VTW Chuan CY Halim SA Ghani ARI. Deep tendon reflex: the tools and techniques. What surgical neurology residents should know. Malays J Med Sci. (2021) 28:48–62. 10.21315/mjms2021.28.2.5 33958960 PMC8075597

[12] Tham LK Abu Osman NA Wan Abas WA Lim KS. The validity and reliability of motion analysis in patellar tendon reflex assessment. PLoS ONE. (2013) 8:e55702. 10.1371/journal.pone.0055702 23409022 PMC3567131

[13] Moore BD Drouin J Gansneder BM Shultz SJ. The differential effects of fatigue on reflex response timing and amplitude in males and females. J Electromyog Kinesiol. (2002) 12:351–60. 10.1016/S1050-6411(02)00032-9 12223167

[14] Morimoto T Hirata H Watanabe K Kato K Otani K Mawatari M. The usefulness of deep tendon reflexes in the diagnosis of lumbar spine diseases: a narrative review. Cureus. (2024) 16:e55772. 10.7759/cureus.55772 38586775 PMC10999014

[15] Campbell A Caldwell J Yalamanchili D Sepanek L Youssefzadeh K Uquillas C. Effect of patient height and sex on the patellar tendon and anterior cruciate ligament. Orthopaedic J Sports Med. (2021) 9:23259671211003244. 10.1177/23259671211003244 34017879 PMC8114262

[16] Pazzinatto MF de Oliveira Silva D Ferreira AS Waiteman MC Pappas E Magalhaes FH. Patellar tendon reflex and vastus medialis hoffmann reflex are down regulated and correlated in women with patellofemoral pain. Arch Phys Med Rehabil. (2019) 100:514–9. 10.1016/j.apmr.2018.06.024 30059658

[17] Kiso Inc. (2024). Available at: https://www.kisoinc.com/ (accessed October 1, 2024).

[18] Thought Technology. Triode Electrodes - *T3402M*. (2022). Available at: https://thoughttechnology.com/triode-electrodes-t3402m/ (accessed September 18, 2024).

[19] Pilkington O Karason H Helgason T. EMG Synchronized Reflex Hammer for precise Patella reflex test. Biomed Eng-Biome Tech. (2021) 66:244. 10.1515/bmt-2021-6035

[20] Kristinsdottir S Thorisdottir AT Halldorsdottir LB Magns~dottir G Ingolfsdottir B Ingvarsson PE. A novel reflex analysis of healthy and spinal cord-injured individuals. Curr Direct Biomed Eng. (2022) 8:745–8. 10.1515/cdbme-2022-1190

[21] Gustafsson F. Determining the initial states in forward-backward filtering. IEEE Trans Signal Proc. (1996) 44:988–92. 10.1109/78.492552 39635258 PMC11616419

[22] Ward NS. Compensatory mechanisms in the aging motor system. Ageing Res Rev. (2006) 5:239–54. 10.1016/j.arr.2006.04.003 16905372

[23] Takeuchi N Oouchida Y Izumi SI. Motor control and neural plasticity through interhemispheric interactions. Neural Plast. (2012) 2012:823285. 10.1155/2012/823285 23326685 PMC3541646

[24] Goble DJ Coxon JP Van Impe A De Vos J Wenderoth N Swinnen SP. The neural control of bimanual movements in the elderly: Brain regions exhibiting age-related increases in activity, frequency-induced neural modulation, and task-specific compensatory recruitment. Hum Brain Mapp. (2010) 31:1281–95. 10.1002/hbm.20943 20082331 PMC6871108

[25] Reuter-Lorenz PA Jonides J Smith EE Hartley A Miller A Marshuetz C. Age differences in the frontal lateralization of verbal and spatial working memory revealed by PET. J Cogn Neurosci. (2000) 12:174–87. 10.1162/089892900561814 10769314

[26] Péréon Y Nguyen The Tich S Fournier E Genet R Guihņeuc P. Electrophysiological recording of deep tendon reflexes: normative data in children and in adults. Clini Neurophysiol. (2004) 34:131–9. 10.1016/j.neucli.2004.04.005 15501682

